# Reactivation of Cutaneous Leishmaniasis in a Rheumatoid Arthritis Patient Undergoing Tocilizumab Treatment

**DOI:** 10.4269/ajtmh.26-0345

**Published:** 2026-09-15

**Authors:** Regina Maia de Souza, Felipe Francisco Tuon, Antônio Carlos Nicodemo, Christini Takemi Emori, Beatriz Julieta Celeste, Maria Carmen Arroyo Sanchez, Valdir Sabbaga Amato

**Affiliations:** ^1^ Laboratório de Parasitologia, São Paulo, Universidade de São Paulo Instituto de Medicina Tropical de São Paulo, São Paulo, Brazil;; ^2^ Laboratório de Doenças Infecciosas e Emergentes, Pontifícia Universidade Católica do Paraná, Curitiba, Brazil;; ^3^ Faculdade de Medicina, Departamento de Infectologia e Medicina Tropical, São Paulo, Universidade de São Paulo, São Paulo, Brazil;; ^4^ Laboratório de Soroepidemiologia, São Paulo, Universidade de São Paulo Instituto de Medicina Tropical de São Paulo, São Paulo, Brazil

## Abstract

Cutaneous leishmaniasis is a neglected vector-borne disease caused by *Leishmania* spp., with viable parasites potentially persisting after clinical cure. Immunosuppressive therapies may favor reactivation of latent infection. The case of a 62-year-old Brazilian woman with a 25-year history of rheumatoid arthritis who developed a painful ulcerative lesion on her right leg 1 month after initiating tocilizumab therapy is reported. Molecular testing confirmed *Leishmania (Viannia) braziliensis*, consistent with reactivated cutaneous leishmaniasis. Tocilizumab was discontinued, and liposomal amphotericin B was initiated but interrupted because of nephrotoxicity and hepatotoxicity. Despite incomplete treatment, the lesion gradually healed after withdrawal of immunosuppression without requiring additional antiparasitic therapy. Secondary prophylaxis with liposomal amphotericin B was subsequently initiated under close clinical and laboratory monitoring, and no recurrence has been observed during follow-up. This case highlights the importance of screening clinical history for prior leishmaniasis, maintaining vigilance for reactivation in patients from endemic areas, and promptly adjusting immunosuppressive therapy to optimize clinical outcomes.

## INTRODUCTION

Cutaneous leishmaniasis is a group of neglected diseases characterized by a chronic course, which can result in healing lesions that may occur spontaneously or through therapy. This vector-borne disease is caused by various species of the genus *Leishmania*.^1^

The lesions may have viable parasites, including in healed lesions, where these parasites can remain alive for many years within the stable cells of the host. During an effective immune response against *Leishmania*, the most important effector cells are CD4+ T-helper lymphocytes, which can be divided into at least two subpopulations. Elevated levels of cytokines, such as IFN-γ, IL-12, IL-2, and TNF-α, are associated with poor response to the disease. At the same time, the production of IL-4, IL-5, IL-10, and IL-13 can inhibit macrophage activation and contribute to the disease progress.^2^^,^^3^

Rheumatoid arthritis (RA) is a systemic, inflammatory, and autoimmune disease that primarily affects the synovial membrane, especially in peripheral joints. Its prevalence is estimated to be between 0.5% and 1% of the population, with a higher incidence in women ages 30 to 50 years old.^4^ In RA, antigen-activated CD4+ T cells enhance the immune response by stimulating other immune cells, such as mononuclear cells, synovial fibroblasts, chondrocytes, and osteoclasts. The cytokines released, particularly TNF-α, IL-6, and IL-1, contribute to synovial inflammation.^5^ IL-6 is a pleiotropic cytokine capable of modulating both proinflammatory and anti-inflammatory responses in the innate immune system.^6^

The field of RA therapy is rapidly evolving with a focus on using potent immunosuppressive drugs, such as methotrexate, and anticytokine immunobiologics (monoclonal antibodies).^7^ Among these monoclonal antibodies, tocilizumab (TCZ) acts as a competitive inhibitor of IL-6 receptor-mediated signaling, showing promise in controlling RA. However, cases of chronic infection reactivation have been reported in patients undergoing TCZ therapy, particularly among those with mycobacterial diseases.^8^

Immunosuppressive treatments for RA may also increase the risk of reactivation of cutaneous or mucocutaneous leishmaniasis,^9^ especially in patients who were previously infected and migrated from endemic areas. Given that RA typically occurs later in life, previous exposure to cutaneous leishmaniasis often originates from endemic regions, which can lead to difficulties in the management and diagnosis of a new skin lesion during after immunobiological therapy.

We think that this is the first documented case of a patient with recurrent cutaneous leishmaniasis while undergoing treatment with TCZ.

## CASE REPORT

Here, we report a case of RA in a 62-year-old Brazilian woman who had the diagnosis of RA for 25 years. She lived in an endemic area of cutaneous leishmaniasis, and in 2016, she reported the appearance of a cutaneous ulcer on her left ankle; the aspirate of the ulcer revealed the presence of *Leishmania* DNA.

In May 2016, the patient was treated with liposomal amphotericin as recommended by the Ministry of Health of Brazil for immunocompromised patients (https://www.gov.br/saude/pt-br/centrais-de-conteudo/publicacoes/svsa/leishmaniose-tegumentar/leishmaniose-nas-americas-diretrizes-para-o-tratamento; 3 mg/kg per day of a liposomal amphotericin protocol for 10 days for a total cumulative dose of 35 mg) at Hospital das Clínicas da Faculdade de Medicina da Universidade de São Paulo, Brazil, resulting in wound healing.^10^

She was then transferred to the outpatient clinic for immunosuppressed patients at the same institution because of her RA.

In March 2023, the patient began treatment of RA solely with the immunobiologics therapy TCZ administered once a month for 3 months. She did not receive the last infusion of TCZ because she developed a painful ulcerated lesion approximately 4 cm in diameter on the lateral side of her right leg (Figure 1A). During the period of TCZ therapy, the patient reported no travel to endemic regions and no insect bites. Serological tests for fungi, leishmaniasis, HIV, and syphilis were all negative. A biopsy was performed. The stains for Grocott and Ziehl–Neelsen as well as immunohistochemical staining for *Leishmania* and human herpesvirus 8 were negative. *Leishmania* DNA was detected using kinetoplast DNA polymerase chain reaction, and next-generation sequencing identified the species as *Leishmania (Viannia) braziliensis*, which is prevalent in Brazil. Tocilizumab was discontinued (Figure 1B), and treatment with liposomal amphotericin B was initiated, administering a cumulative dose of 20 mg/kg (Figure 1C). This treatment was temporarily suspended in July 2023 because of nephrotoxicity and hepatotoxicity. As a result, the lesion remained ulcerated until September 2024 (Figure 1D), when it completely healed after stopping immunosuppressive therapy with TCZ without the need for retreatment with liposomal amphotericin B (Figure 1E). At the beginning of 2025, the patient began secondary prophylaxis with liposomal amphotericin B at a dose of 3 mg/kg every 21 days, and the lesion remained healed.

**Figure 1.**
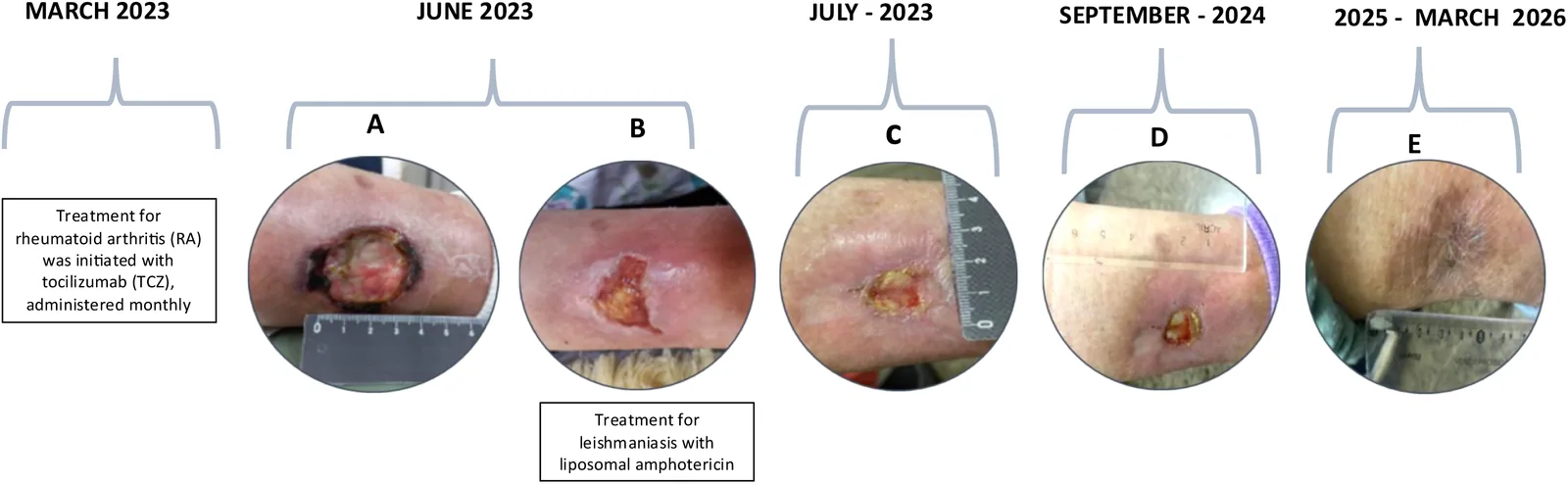
(**A**) Lesion during treatment with tocilizumab (TCZ). A painful ulcerative lesion approximately 4 cm in diameter developed on the lateral aspect of the right leg 2 months after initiation of TCZ therapy. Diagnostic evaluation revealed negative serological tests for fungal infections, HIV, syphilis, and leishmaniasis. Histopathological examination, including special stains for fungi and mycobacteria, human herpesvirus 8 testing, and immunohistochemistry for *Leishmania*, was negative. Nevertheless, polymerase chain reaction targeting kinetoplast DNA was positive for *Leishmania* spp. (**B**) Lesion after discontinuation of TCZ. Tocilizumab was discontinued, and treatment with liposomal amphotericin B was initiated on June 19, 2023, with a planned cumulative dose of 20 mg/kg. (**C**) Liposomal amphotericin B was temporarily interrupted because of nephrotoxicity and hepatotoxicity. Treatment was completed on July 6, 2023. (**D**) Therapeutic failure of liposomal amphotericin B. (**E**) Complete healing after discontinuation of TCZ.

## DISCUSSION

Control of *Leishmania* infection primarily relies on cellular immunity, which directs the immune response to the sites of infection. Consequently, cytokines and chemokines play crucial roles in this immune response, which can be influenced by various factors, such as the host’s genetic background, malnutrition, and immune suppression.^10^^,^^11^

*Leishmania* has developed several strategies to evade the host’s immune defenses, including 1) inhibiting the host cell’s ability to present the parasite’s antigens to other components of the immune system by interfering with the expression of major histocompatibility complex class II molecules and reducing the expression of costimulatory molecules, such as B7-1 and CD40; 2) suppressing the production of proinflammatory cytokines (IL-1, TNF-α, and IL-6) and cytokines that activate T lymphocytes (IL-12); and 3) inducing immunosuppressive molecules, like prostaglandin E2, TGF-β, and IL-10.^3^

Proinflammatory cytokines, including TNF-α, IFN-γ, IL-1, IL-2, IL-8, IL-12, IL-15, IL-18, and IL-17, are mainly produced to enhance the immune response to *Leishmania* infection. In contrast, anti-inflammatory cytokines, such as IL-5, IL-6, IL-4, IL-10, IL-13, and TGF-β, serve as immunoregulatory molecules that counteract the effects of proinflammatory cytokines, limiting inflammation caused by their excessive production.^12^

In addition to the Th1/Th2 paradigm, the cytokine IL-17, which is secreted by activated CD4+ T cells (Th17 cells) and neutrophils, has been implicated in leishmaniasis and several significant inflammatory human diseases.^12^

In RA, the release of TNF-α, IL-6, IL-1, and IL-17 by antigen-activated CD4+ T cells leads to synovial inflammation. IL-17 secreted by Th17 cells can stimulate macrophages to produce inflammatory cytokines, matrix metalloproteinases, and nitric oxide, contributing to inflammatory infiltrate and cartilage damage. It may also amplify the immune response by stimulating other mononuclear cells, synovial fibroblasts, chondrocytes, and osteoclasts.^5^^,^^13^

Tocilizumab, an anti-IL-6 agent, has been approved for the treatment of RA, giant cell arteritis, juvenile idiopathic arthritis, Castleman disease, and other immune-mediated diseases.^14^ However, there has been a reported risk of infections associated with its use.^8^

Studies have shown that even after a year of clinical healing, patients’ scars still exhibit microscopically clear signs of inflammatory activity, which can lead to early relapses of the infection. Significant decreases in the local inflammatory process were only observed after 3 years of clinical cure of the lesions.^15^ Thus, patients with RA undergoing immunosuppressive therapy are at risk for leishmaniasis reactivation.^9^^,^^16^

One of the escape mechanisms used by *Leishmania* is the inhibition of cytokines involved in the proinflammatory response, including IL-1, TNF-α, and IL-6. In the present report, the inhibition of IL-6 receptor production was intensified with the use of TCZ, disrupting the balance of the inflammatory response and potentially reactivating latent *Leishmania*. This IL-6 cytokine exhibits pleiotropic characteristics, functioning as both a proinflammatory mediator and an anti-inflammatory mediator.

A meta-analysis conducted by Campbell et al.^17^ reported that the main infections observed in patients using TCZ were skin and soft tissue infections, respiratory tract infections, and the reactivation of tuberculosis or viral hepatitis. In this meta-analysis, a small but statistically significant increase in adverse events and infections was observed among patients treated with TCZ at a dose of 8 mg/kg compared with the control group. However, this risk was comparable with that associated with other biologic agents, whereas the risk of serious infections appeared to be lower than that reported for TNF inhibitors.

Neoplasms have also been reported, potentially occurring in patients with chronic autoimmune disorders that are intrinsically associated with the activation of autoreactive T and B lymphocytes and the release of proinflammatory cytokines, which may increase the risk of cancer.^18^^,^^19^

The dose reduction strategy may represent a reasonable alternative to minimize the risk of infectious complications while maintaining control of the underlying rheumatic disease. IL-6 plays a key role in the immune response to *Leishmania* infection. In combination with TGF-β, it promotes the differentiation of naïve T cells into Th17 cells, which produce IL-17 and contribute to the recruitment of effector cells to the site of infection. This pathway may enhance local parasite control; therefore, IL-6 blockade could potentially impair protective immune mechanisms involved in the containment of *Leishmania* infection.^12^ The frequency of relapse associated with the reintroduction of immunosuppressive agents is still poorly understood as this issue has received limited attention in the literature. Furthermore, there is currently no consensus regarding whether immunosuppressive therapy should be discontinued, whether immunosuppressive therapy should be dose reduced, or when it should be safely resumed after leishmaniasis treatment.

Evidence regarding the management of TNF-α inhibitors during leishmaniasis remains limited. In a case series of patients with cutaneous leishmaniasis receiving TNF-α antagonists, anti-TNF therapy was discontinued in most cases after the diagnosis of leishmaniasis. However, the authors concluded that there are insufficient data to recommend routine discontinuation of TNF-α antagonists during or after antileishmanial treatment. They emphasized that the risk of progression of the underlying rheumatic disease should be carefully balanced against the risk of persistent or recurrent infection. After complete resolution of the cutaneous lesions, TNF-α antagonist therapy may be resumed at the lowest effective dose under close clinical monitoring.^20^

Although secondary prophylaxis is not routinely recommended after clinical cure of cutaneous leishmaniasis, viable parasites may persist within healed lesions, potentially serving as a source of reactivation in immunosuppressed individuals. Clinical cure of cutaneous leishmaniasis does not necessarily imply sterile eradication of the parasite.^21^

In our patient, prophylactic treatment was instituted after the relapse as an individualized, empirical decision based on the occurrence of multiple recurrences and the presumed persistence of the parasite. This approach was adopted after careful consideration of the potential risks and benefits, recognizing the lack of supporting evidence and the uncertainty regarding the optimal duration of prophylaxis.

Remarkably, the patient was considered cured after discontinuing immunosuppressive therapy without the need for retreatment with liposomal amphotericin B.

## CONCLUSION

The use of an IL-6 inhibitor (TCZ) has been useful in the treatment of RA. However, it can disrupt the immune system’s ability to respond to infections, such as leishmaniasis. This serves as a caution for physicians regarding the potential risk of reactivating latent leishmaniasis in immunosuppressed patients. We draw the attention of physicians who deal with the treatment of RA to the possibility of reactivation of latent infectious agents during the immunosuppression phase caused by immunobiological agents, especially in areas where cutaneous leishmaniasis is endemic or has a large number of migrants from endemic areas.
